# CO_2_ Laser therapy versus cryotherapy in treatment of genital warts; a Randomized Controlled Trial (RCT)

**Published:** 2012-12

**Authors:** M Azizjalali, GH Ghaffarpour, B Mousavifard

**Affiliations:** 1 Skin and Stem Cell Research Center, Tehran University of Medical Sciences, Tehran, Iran; 2 Department of Dermatology, Rasoul Akram Hospital, Tehran University of Medical Sciences, Tehran, Iran

**Keywords:** Genital warts, Human Papillomavirus, CO_2_ laser, cryotherapy

## Abstract

**Background and Objective:**

Genital warts, due to specific types of the human papillomavirus, have long been regarded as one of the most important causes of cervical cancer and one of the most common cause of Sexually Transmited Diseases (STDs). Over the years, it has been the focus of several studies in order to find the best effective approach to eradicate the virus, however there are still controversies regarding this matter. We compared efficacy of the two physical ablative therapies; Liquid Nitrogen and CO_2_ laser.

**Material and Methods:**

One hundred and sixty patients, with external genital warts, were divided into two groups. Each group consisted of 80 patients treated with CO_2_-laser or cryotherapy. Clearance and recurrence rates were evaluated for 3 months.

**Results:**

Complete clearance was achieved in 76 lesions (95%) treated by CO_2_-laser and 37 lesions (46.2%) treated by cryotherapy which was significantly different (p < 0.001). In the CO_2_-laser group, lesions required only one treatment to clear while in the cryotherapy group, lesions required two (12%) even up to three (12.2%) treatments for some patients to clear completely. Laser therapy was associated with less recurrence rate compared to cryotherapy (0.05% Vs 0.18%).

**Conclusion:**

Generally, the efficacy of CO_2_ laser treatment of external genital warts was approximately two fold greater than cryotherapy and it was associated with lower recurrence rate.

## INTRODUCTION

Human Papilloma Viruses (HPVs) comprise a large group of approximately 120 genotypes that infect the epithelia of the skin or mucosa and most commonly cause benign Papillomas. Anogenital HPV is a highly prevalent sexually transmitted infection by more than 40 types of HPV, seen predominantly in young adults (1–4).

Condyloma accuminatum or benign anogenital warts are typically caused by HPV-6 or 11 which are considered low-risk types. Persistent infection with high-risk HPV types predominantly HPV-16 and 18 (5 and 7) is the primary and major cause of cervical cancers and a subset of vaginal, vulvar, penile, anal, oropharyngeal and rarely squamous cell carcinoma of the digits (8).

Genital warts are associated with adverse psychological effects resulting to decreased quality of life and increasing medical care costs (9–10). At present there is no specific antiviral therapy available to cure HPV infection (11). Destructive or ablative therapies for genital warts include cryotherapy, Trichloracetic acid (TCA), electrosurgery, Currettage, Scalpel or scissors excision, laser vaporization and photodynamic therapy with topical aminolevulinic acid (12–13).

## MATERIALS AND METHODS

### Study population

This prospective, randomized study was conducted to compare the effect of cryotherapy and CO_2_ laser therapy in treatment of genital warts. Between November 2009 and December 2010, all patients with documented lower genital warts who visiting dermatology clinic in Hazarat Rasool Akram hospital in Tehran, Iran, were screened for eligibility and 160 patients were selected for this study. The inclusion criteria consisted of lesions with diameter of equal or more than 10 mm, which located on the pubis, penis, scrotum, vulva or inguinal area. Vaginal and cervical lesions were not considered for this study. Exclusion criteria were history of immunosuppressive status, history of immune modulator drugs use that were administered in the past four weeks, history of local antiviral agents use in the past two weeks, pregnancy, breast-feeding, destructive therapies or presence of any other concomitant STD. A total of 160 patients which enrolled in the study were equally allocated into two groups using a computerized random number. One group underwent CO_2_ laser therapy and the other group was treated with cryotherapy. The study group and the type of treatment has been blinded to the dermatologists who examined the patients after treatment to evaluate the lesions. Characteristics of the lesions comprising duration of the disease, as well as location of the lesions were also evaluated. Patients in both groups were informed with informations about the etiology of genital warts, natural history of the disease, its complications, treatment choices and prevention methods. They were also informed that both treatment choices were academic and classic methods for treatment of the genital warts. A written informed consent obtained from patients. The Islamic ethics committee of Tehran University of Medical Sciences approved the study. In the cryotherapy group, there was no need for local anesthesia.

### CO_2_ laser and Crayotherapy treatment

In the laser therapy group, after routine decontamination of the lesions’ area and local anesthesia, the wart and a 2 mm surrounding margin of normal skin were evaporated in focal distance of laser light using unixel CO_2_ laser unit, 30 watt fluence, made in south Korea with continuous mode wavelength of 10600 nm with fluency of 4.5 J/cm2. In cryotherapy method, the wart and 2 mm of the normal surrounding margin were frozen using liquid nitrogen -196°C and open spray mode in two freeze/thaw cycles. After lesion removal, tetracycline ointment was applied on the area for 24 hours.

### Treatment evaluation

Lesions were evaluated after 2 weeks and then three months later. Second and third applications were performed every two weeks to completely clear the lesions. Effectiveness index was defined as complete clearance of lesions and was calculated as the number of cleared lesions divided by total number of lesions multiplied by 100.

### Statistical analysis

Basic characteristics were described by mean values and standard deviations. The univariable analyses of the continuous and categorical variables were carried out using t-tests and chi-square tests. A p-value of < 0.001 was considered significant. Statistical analysis was performed using SPSS 16 for Windows (SPSS Inc., Chicago, Illinois).

## RESULTS

Table. 1 shows basic characteristic of the lesions in the two groups. In CO_2_ laser group, 76 lesions (95%) and in cryotherapy group, 37 lesions (46.2%) were completely eradicated respectively. The observed difference was statistically significant, (p < 0.001). In cryotherapy group, second and third treatment were needed for complete treatment in 12% and 12.2% of lesions, respectively, while in CO_2_ laser therapy, all lesions showed clearance after a single treatment. Post procedure hypopigmentation was not statistically different two groups. Blistering developed in 2 lesions of cryotherapy group (p = 0.99) and non of cases in laser group. Four lesions recurred in CO_2_ laser therapy group (2.5%) and 7 lesions in cryotherapy group (18%).

**Table 1:** Basic characteristics of the lesions and treated groups.

	CO_2_ laser therapy (N = 80)	Cryotherapy (N = 80)
Duration of disease (%)
≤4 months	61 (76.2%)	56(70%)
≥9 months	1 (1.2%)	1(1.2%)
Location of lesions (%)
Penis	18(22.5%)	14(17.5%)
Scrotum	23(28.7%)	28(35.0%)
Vulva	22(27.5%)	19(23.7%)
Inguinal	17(21.2%)	19(23.7%)

Data are presented as (%) and mean ± standard deviation.

## DISCUSSION

Different treatment modalities have been described for genital warts, all of them associated with advantages and disadvantages (13–16). The appropriate treatment modality depends on the number, size, and location of the lesions in addition to patient's immunologic status. The ideal treatment is the one that could clear the lesions completely with a minimal amount of pain, hypo or hyperpigmentation, scars, local and systemic adverse effects in addition to lower recurrence rates that are mostly achieved by physical ablative therapies when compared to medication therapies. CO_2_ laser and cryotherapy are of those physical ablative treatment methods with less adverse effects and high clearance rates (13). Cryotherapy is one of the most effective treatments available for genital warts with clearance rates of about 79-88% (17). It is an inexpensive, easy applicable method with rapid destructive effect and no serious systemic side effects that make it a safe therapy in pregnancy. Although, it has some limitations such as risk of vaginal perforation in case of using liquid nitrogen, recurrence in lesions larger than the cryoprobe and some local side effects including blistering and local necrosis (18). However, hypopigmentation and scar formation are reported rarely (18). Healing usually occurs in 1-2 weeks time after cryotherapy, although sometimes complete healing may take more than 2 weeks. Lasers are more complex and costly in comparison to other treatment options for genital warts, however mostly used in refractory external genital lesions (18). It is commonly used in treatment of anorectal, penile, and urethral warts in men and cervical and flat vaginal warts in women. Laser therapy needs local anesthesia in most of the cases however it can be used in extensive and thick lesions effectively as it is able to penetrate the lesions deeper than generally occurs with cryotherapy (17). Due to the aforementioned reasons in addition to its painless act, rapid healing, limited rate of complication, infection and recurrence rates, and minimal risk of scarring, it could be considered as a safe and effective therapeutic method for genitalia warts even in childhood and pregnancy (19). Its adverse effects include pruritus, hypopigmentation and scar formation. A disadvantage of the CO_2_ laser in comparison with cryotherapy is greater risk of scarring. In our study, 95% of lesions treated with CO_2_ laser were cleaned in a single session, while 46.2% of lesions in cryotherapy group were cleaned after 3 sessions during a 2-week interval. Cryotherapy is a simpler procedure, requiring a liquid nitrogen tank and a spray, while CO_2_ laser requires laser machine. In this study, most lesions treated with cryotherapy were cleaned in multiple therapeutic sessions in 2-week intervals, while CO_2_ laser therapy is basically a once-only treatment. Former trials showed recurrence rate of 25-39% when genital warts removed by cryotherapy and 60 to 77% when treated by CO_2_ laser (17–20). However, given the result of our study, treating with CO_2_ laser is associated with less recurrence rate than cryotherapy (0.05% against 0.18%). In conclusion, CO_2_ laser is more effective than cryotherapy (about two folds) in treatment of external genital warts due to its clearance, recurrence and complication rates.
